# Prostate cancer research on social media platforms: a bibliometric and thematic analysis

**DOI:** 10.3389/fonc.2026.1799912

**Published:** 2026-06-02

**Authors:** Yang Xie, Huining Huang, Xiurui Chen, Lihua Chen, Zhenning Guo

**Affiliations:** 1 Department of Urology, The Second Nanning People’s Hospital & The Third Affiliated Hospital of Guangxi Medical University, Nanning, China; 2 College of Mechanical and Electronic Engineering, China University of Petroleum (East China), Qingdao, China; 3 Department of Clinical Laboratory Medicine, The First People’s Hospital of Nanning & The Fifth Affiliated Hospital of Guangxi Medical University, Nanning, China; 4 School of Foreign Studies, China University of Petroleum (East China), Qingdao, China

**Keywords:** disease management, information management, patient education, prostate cancer, social media

## Abstract

**Introduction:**

Prostate cancer is a highly prevalent malignancy among men globally, posing significant challenges in prevention, treatment, and patient management. Social media platforms, with their real-time accessibility, broad reach, and interactive features, offer novel data sources and intervention avenues for prostate cancer research. However, current studies in this field remain fragmented, lacking a systematic synthesis of the overall knowledge structure, developmental trajectory, and collaborative networks.

**Methods:**

A comprehensive search of Web of Science (WoS), PubMed, and IEEE Xplore databases was conducted to identify studies related to prostate cancer on social media platforms published between 2010 and 2025. Eligible studies were analysed using VOSviewer and R package bibliometrix to evaluate publication trends, authorship, citations, and thematic focus.

**Results:**

The search retrieved 483 studies, of which 249 met the inclusion criteria. These studies involved 1, 649 authors and were published in 155 journals. A significant increase in publications was observed from 2017 to 2025, with notable citation peaks in 2010, 2014, and 2019. Citation pathways and highly cited publications further elucidated the field’s evolution. The United States contributed the most publications (n=52), while the United Kingdom led in international collaborations (Total Link Strength=66). High-frequency keywords included ‘quality-of-life’, ‘information’, ‘radical prostatectomy’, ‘risk’, ‘Twitter’ and ‘communication’. Three major thematic areas were identified: (1) health information quality assessment, (2) patient education, and (3) personalized patient support and quality-of-life interventions. A strong correlation between prostate cancer and breast cancer research on social media was also observed. Thematic evolution revealed a shift from early foundational biological studies to patient-centred psychosocial support, and further toward integrating artificial intelligence for disease trend monitoring, prognosis prediction, and quality-of-life interventions.

**Conclusion:**

This study presents the first systematic bibliometric analysis of prostate cancer research on social media platforms, outlining the field’s knowledge map and developmental trajectory. The findings highlight a paradigm shift from micro-level individual care to macro-level precision monitoring, offering evidence-based insights for researchers, healthcare professionals, and policymakers. Future efforts should prioritize interdisciplinary collaboration, optimize information quality, promote equitable access to health information, and integrate emerging technologies into health communication and public health research.

## Introduction

Prostate cancer is one of the most common malignant tumours among men. According to the 2020 data from the International Agency for Research on Cancer (IARC), there were 1.414 million new cases and 375, 000 deaths worldwide, ranking second and fifth among male cancers in incidence and mortality, respectively (1). With advances in diagnostic and therapeutic technologies, patient survival rates have significantly improved. However, the symptom burden, psychological distress, and information needs associated with long-term survival have become increasingly prominent. During the course of the disease, patients may experience physical suffering caused by surgery, radiotherapy, chemotherapy, and endocrine therapy, while disease progression can also impair quality of life (2). Traditional hospital-centred cross-sectional survey and intervention models are unable to address patients’ multidimensional needs throughout the entire disease trajectory (3). Therefore, exploring novel approaches to the pathogenesis, treatment methods, and disease management of prostate cancer is of great importance for improving patient survival and quality of life, as well as reducing the burden on families and society.

Social media platforms, characterized by real-time communication, anonymity, and interactivity, are playing an increasingly important role in the healthcare field and have provided new opportunities for prostate cancer research (4). These platforms aggregate vast amounts of user-generated content (UGC), including patients’ symptom descriptions, treatment experience, and recovery records (5), thereby offering valuable data resources for medical research. Analysis of such information can provide insights into disease trends, symptom characteristics, and feedback on treatment outcomes (6, 7).

Social media has also expanded the boundaries of doctor-patient communication. Patients can consult healthcare professionals at any time to obtain disease-related guidance, while clinicians can use these platforms to disseminate disease knowledge, share treatment strategies, and provide rehabilitation recommendations, thereby enhancing patient adherence and self-management abilities (1, 8). In addition, peer support and communication among patients may help alleviate psychological distress (9). In the fields of medical education and health communication, social media can transform complex medical information into knowledge that is easier for the public to understand through text, images, videos, and live broadcasts, thereby improving health literacy and disease prevention awareness. For example, disseminating information on early symptoms, screening methods, and treatment options for prostate cancer may facilitate early diagnosis and intervention (10, 11).

In recent years, scholars have increasingly utilized social media to conduct prostate cancer-related studies, including the use of natural language processing to analyse patient forums for identifying unmet needs (12), evaluating the impact of public health events on diagnostic and treatment pathways (13), and assessing the effects of health information interventions on patient decision-making and psychological well-being (14). However, these studies have largely focused on single platforms or isolated topics, lacking integration into a coherent body of knowledge and making it difficult to comprehensively characterize the research landscape and emerging trends in this field.

Based on the above background, this study aims to systematically review research on prostate cancer and social media through bibliometric and thematic analyses, with a focus on the following questions:

- What are the publication trends, core journals, and leading researchers in the field of prostate cancer research on social media?
- What are the main research themes, and how have they evolved over time?
- How does international collaboration influence research output and knowledge dissemination?
- What are the emerging frontiers and future directions in research and practice?

By addressing these questions, this study aims to provide researchers, healthcare professionals, and health information platform developers with an evidence-based overview to better understand the current applications and future potential of social media in prostate cancer information dissemination, patient support, and health interventions.

## Methods

### Data sources and search strategy

The literature search was conducted on December 20, 2025. The search query consisted of two parts connected by the Boolean operator ‘AND’: the first part integrated terms related to prostate cancer, and the second part encompassed terms related to social media platforms and associated concepts. To ensure comprehensive coverage of relevant literature from multidisciplinary perspectives, including medical, information science, and sociological fields, this study incorporated three databases: WoS, PubMed, and IEEE Xplore. WoS is a core multidisciplinary citation index database from Clarivate. It’s renowned for rigorous journal selection criteria, indexing high-quality, high-impact academic journals globally, and providing powerful citation analysis capabilities (15). PubMed, developed and maintained by the US National Library of Medicine (NLM), is the most authoritative and comprehensive free literature database in the biomedical field (16). IEEE Xplore is provided by the Institute of Electrical and Electronics Engineers (IEEE), which is an authoritative database for computer science, information technology, electronic engineering, and related fields, containing a vast collection of high-quality journals, conference proceedings, and technical standards (17). The specific search strings were adjusted according to the syntax rules of each respective database (**Table 1**).

**Table 1:** Search strategies used for each database.

Database	Search query
Web of Science	TS=((“prostatic neoplasms” OR “prostate cancer” OR “prostatic cancer” OR “carcinoma of prostate” OR “prostate carcinoma” OR “prostatic adenocarcinoma” OR “adenocarcinoma of prostate” OR “prostate neoplasm” OR “malignant neoplasm of prostate” OR “prostate malignancy”)AND(“social media” OR “online platform*” OR Twitter OR Facebook OR Instagram OR YouTube OR TikTok OR LinkedIn OR Reddit OR WhatsApp OR WeChat OR Telegram OR blog* OR microblog* OR “online communit*” OR “digital communit*” OR “online social network*” OR “digital social network*”))
PubMed	((“prostatic neoplasms”[Title/Abstract] OR “prostate cancer”[Title/Abstract] OR “prostatic cancer”[Title/Abstract] OR “carcinoma of prostate”[Title/Abstract] OR “prostate carcinoma”[Title/Abstract] OR “prostatic adenocarcinoma”[Title/Abstract] OR “adenocarcinoma of prostate”[Title/Abstract] OR “prostate neoplasm”[Title/Abstract] OR “malignant neoplasm of prostate”[Title/Abstract] OR “prostate malignancy”[Title/Abstract]) AND (“social media”[Title/Abstract] OR “online platform*”[Title/Abstract] OR “Twitter”[Title/Abstract] OR “Facebook”[Title/Abstract] OR “Instagram”[Title/Abstract] OR “YouTube”[Title/Abstract] OR “TikTok”[Title/Abstract] OR “LinkedIn”[Title/Abstract] OR “Reddit”[Title/Abstract] OR “WhatsApp”[Title/Abstract] OR “WeChat”[Title/Abstract] OR “Telegram”[Title/Abstract] OR “blog*”[Title/Abstract] OR “microblog*”[Title/Abstract] OR “online communit*”[Title/Abstract] OR “digital communit*”[Title/Abstract] OR “online social network*”[Title/Abstract] OR “digital social network*”[Title/Abstract]))
IEEE Xplore	(“All Metadata”:”prostate cancer” OR “All Metadata”:”prostatic cancer” OR “All Metadata”:”prostate neoplasm*” OR “All Metadata”:”prostatic neoplasm*” OR “All Metadata”:”prostate carcinoma*” OR “All Metadata”:”prostatic adenocarcinoma*” OR “All Metadata”:”adenocarcinoma prostate”) AND (“All Metadata”:”social media” OR “All Metadata”:”online platform*” OR “All Metadata”:Twitter OR “All Metadata”:Facebook OR “All Metadata”:Instagram OR “All Metadata”:YouTube OR “All Metadata”:TikTok OR “All Metadata”:LinkedIn OR “All Metadata”:Reddit OR “All Metadata”:WhatsApp OR “All Metadata”:WeChat OR “All Metadata”:Telegram OR “All Metadata”:blog* OR “All Metadata”:microblog* OR “All Metadata”:”online communit*” OR “All Metadata”:”digital communit*” OR “All Metadata”:”online social network*” OR “All Metadata”:”digital social network*”)

### Study selection and flowchart

**Supplementary Figure 1** illustrates the study selection process flow diagram. Studies were screened according to eligibility criteria. The publication date was restricted from January 1, 2010, to December 20, 2025, to encompass research from the era of social media prominence and reflect the most recent advances. Only peer-reviewed academic articles published in English were included; non-research publications such as conference abstracts, letters, and commentaries were excluded to minimise information bias and ensure the analysis was grounded in complete research studies.

The screening process involved two stages. Initially, two independent authors (YX and XC) assessed the titles and abstracts of all retrieved records to exclude obviously irrelevant literature. Subsequently, full-text articles for the remaining records were obtained and independently evaluated by the same two reviewers to determine their final eligibility based on the inclusion criteria. Any disagreements between the reviewers during either stage were resolved through discussion. If a consensus could not be reached, a third author (ZG) was consulted for adjudication. To ensure the comprehensiveness of the dataset, the final list of included studies was cross-verified with highly cited articles in Google Scholar. The fact that all articles were identified within the search metadata confirms the inclusivity of the search strategy. Furthermore, the high overlap between the identified active journals and prolific authors in this field reinforces the credibility of the search methodology, thereby ensuring the accuracy and objectivity of the study selection. The studies that ultimately met the inclusion criteria proceeded to the subsequent data extraction and analysis phase.

### Data export and cleaning

Records were exported in plain text format, including complete records and cited references. Standardized processing of the raw literature metadata was performed. Using the dplyr and stringr packages in R, the capitalization and format of author and journal names were unified, and publication years were extracted and verified. After cleaning and preprocessing, the standardized data were imported into the bibliometric analysis tools VOSviewer and the R package bibliometrix for subsequent network construction and visual analysis.

### Data analysis and visualisation

This study utilised the R package bibliometrix (version 4.0) and VOSviewer (version 1.6.18) for analysis. Collaboration networks were constructed using VOSviewer 1.6.18. Node identification employed the ‘full counting’ mode, which does not differentiate node weights. To mitigate network distortion caused by large-scale collaborations, the option to ‘Ignore documents co-authored by a large number of organisations/countries’ was selected. Referring to existing studies (18), the thresholds were set to include documents with a maximum of 25 distinct authors and 5 countries per publication. Meanwhile, the minimum publication threshold of ≥2 documents was applied for node inclusion, aiming to exclude occasional authors or institutions from interfering with the overall network structure. For connectivity assessment, isolated nodes were excluded from the final visualisation. The link strength threshold was set to a minimum co-occurrence strength of 1, and the visualisation was limited to the top 1, 000 strongest links to ensure readability of the visualisation.

Research hotspot analysis was performed using the R package bibliometrix (version 4.0) to generate multi-dimensional science mapping of the collected literature. Specifically, the word cloud was generated based on the frequency statistics of Author Keywords. To prevent inevitable and analytically meaningless over-representation, keywords explicitly mentioned in the search strategy (namely, ‘prostate cancer’ and ‘social media’) were excluded from this analysis. All remaining author keywords were extracted and ranked in descending order of frequency. The top 70 high-frequency keywords were selected as the objects for visualisation. The ‘wordcloud()’ function within R package bibliometrix was employed with min.freq = 1 to ensure all selected keywords were included. In the resulting figure, the font size of each keyword is directly proportional to its absolute frequency within the literature corpus, thereby providing an intuitive reflection of the core terminology receiving the closest attention within the field.

The document citation map was generated using the ‘Historiograph’ function within the Intellectual Structure tool of the Bibliometrix R package. The minimum citation threshold was set to 20 to ensure that the analysis focused on publications with relatively strong academic influence. Nodes are coloured by citation cluster. The direction of the arrows indicates the direction of citation: an arrow points towards a foundational document, indicating its being cited by subsequent work, while the arrow tail represents the citing document. Multiple arrows pointing to the same document indicate distinct citation branches, allowing for further tracing of knowledge flow. Documents within the same colour cluster belong to the same knowledge domain.

Keyword cluster analysis was conducted based on Keywords Plus. First, a keyword co-occurrence matrix was constructed. Referring to existing studies (19), the walktrap algorithm was employed to cluster semantically closely related keywords. The walktrap algorithm captures the local structure of the network through random walks and yields natural, interpretable clusters via hierarchical clustering, making it highly suitable for sparse and semantically dense keyword co-occurrence networks. The normalization was set to ‘association’, with the number of nodes set to 50, the minimum number of edges set to 2, and the repulsion force set to 0.1 to enhance visualization clarity. In the resulting network, nodes represent keywords, and their size is determined by Eigenvector Centrality. The thickness of the connecting lines indicates the co-occurrence strength between keywords. Different coloured cluster modules represent distinct research thematic areas.

Factor analysis was conducted by extracting the top 50 high-frequency keywords, in order to balance the stability and interpretability of the factor model while covering the core concepts of the research field (20). The elbow method was used to determine the optimal number of factors. The minimum number of factors at which the increase in explained variance showed a clear inflection point (elbow) and the Silhouette score was ≥ 0.7 was selected, so as to balance model parsimony and interpretability. In the visualisation, node size represents the absolute value of the keyword’s loading on the corresponding factor, and colours differentiate the various factors.

The thematic map was generated using the Leiden algorithm to cluster research themes, in order to avoid isolated or fragmented clusters, thereby enhancing the scientific rigor and visualization clarity of the thematic map. Referring to existing studies (21), the number of keywords was set to 300, and the minimum cluster frequency threshold per 1, 000 documents was set to 5. For each cluster, the five most frequent keywords were displayed, and the community repulsion force was set to 10 to balance cluster compactness and distinguishability. Themes were positioned into four quadrants based on their Density and Centrality: low density-low centrality (emerging or declining themes), low density-high centrality (basic themes), high density-low centrality (highly developed but isolated niche themes), and high density-high centrality (motor themes). This analysis was implemented using the thematicMap() function, systematically revealing the distribution characteristics and development potential of research hotspots.

The analysis of keyword evolution trends was performed using a time-slicing technique, with one year as the time window. A Burst Detection algorithm was applied to identify keywords whose frequency increased significantly within different time periods. The ‘topicEvolution()’ function in R package bibliometrix was used to generate the evolution map. Using Bibliometrix’s default settings, the word minimum frequency was set to 3, and the number of words per year was set to 3. The x-axis represents time, and the y-axis shows the evolution path of keywords or themes, thereby identifying the dynamic evolution of research hotspots and the shifting trajectory of research frontiers in the field.

## Results

### Publication and citation growth trends

The study identified a total of 249 publications in the field of social media-based prostate cancer research, published between 2010 and 2025. These publications originated from 155 distinct sources. The annual publication output in this field demonstrated a consistent growth trend. The body of literature involved 1, 649 authors and garnered a total of 5, 171 citations. The most frequently cited single publication received 486 citations, and the average number of citations per article was 20.77, indicating sustained academic interest in this research area.

**Supplementary Figure 2** illustrates the temporal trends in the annual number of publications and citation metrics for literature related to prostate cancer research on social media platforms from 2010 to 2025. Regarding publication volume, the annual number of articles showed a general fluctuating upward trend, increasing from 2 publications in 2010 to 29 in 2025. Notable increases were observed in 2018 (24 articles), 2020 (22 articles), and 2021 (32 articles), with the peak within the observation period reached in 2021 (32 articles). Concerning citation impact, the mean total citation count per article (MeanTC/article) exhibited considerable fluctuation. Publications from 2010 had the highest MeanTC/article (214.50), followed by those from 2019 (71.20), suggesting that research outputs from these years attracted closer academic attention. In contrast, the mean total citation count per year (MeanTC/year) showed a clear decreasing trend over time. This metric was higher for earlier years (eg, 2010) and significantly lower for more recent years (eg, 0.62 for 2025), which is attributable to the shorter time available for citations to accumulate. The consistent growth in publication numbers, combined with the dynamic changes in citation metrics, collectively reflects the growing research vitality of this field, with certain years producing influential key findings.

### Analysis of high-yield entities

**Table 2** summarizes the most productive journals, countries/regions, and authors in the field of social media-based prostate cancer research. Among the 249 included publications, the distribution of academic journals revealed that BMJ Open, BJU International, and European Urology were the leading sources, each publishing 7 articles (2.81%). At the country/region level, the United States was the dominant contributor, producing 52 publications (20.88%), substantially surpassing other nations. The United Kingdom and Canada followed, with 22 (8.84%) and 21 (8.43%) articles, respectively. Regarding individual author productivity, Loeb S was the most prolific contributor, with 7 publications (2.81%).

**Table 2:** Top-ranked journals, countries/regions, and authors by publication output in prostate cancer research on social media platforms.

Sources	Articles	Countries/regions	Articles	Authors	Articles
BMJ OPEN	7	USA	52	LOEB S	7
BJU INTERNATIONAL	7	ENGLAND	22	BYRNE N	5
EUROPEAN UROLOGY	7	CANADA	21	BORGMANN H	4
AMERICAN JOURNAL OF MEN’S HEALTH	5	AUSTRALIA	16	BRIGANTI A	3
JOURNAL OF GLOBAL ONCOLOGY	5	CHINA	14	Walter D	3
CUREUS	4	ITALY	12	KATZ MS	3
DIGITAL HEALTH	4	GERMANY	11	SALEM J	3
JOURNAL OF CANCER EDUCATION	4	NETHERLANDS	9	AHMED HU	2
JOURNAL OF SEXUAL MEDICINE	4	FRANCE	9	BENDER JL	2
JOURNAL OF UROLOGY	4	SPAIN	6	BOORJIAN SA	2

### Collaboration network analysis

**Figure 1:**
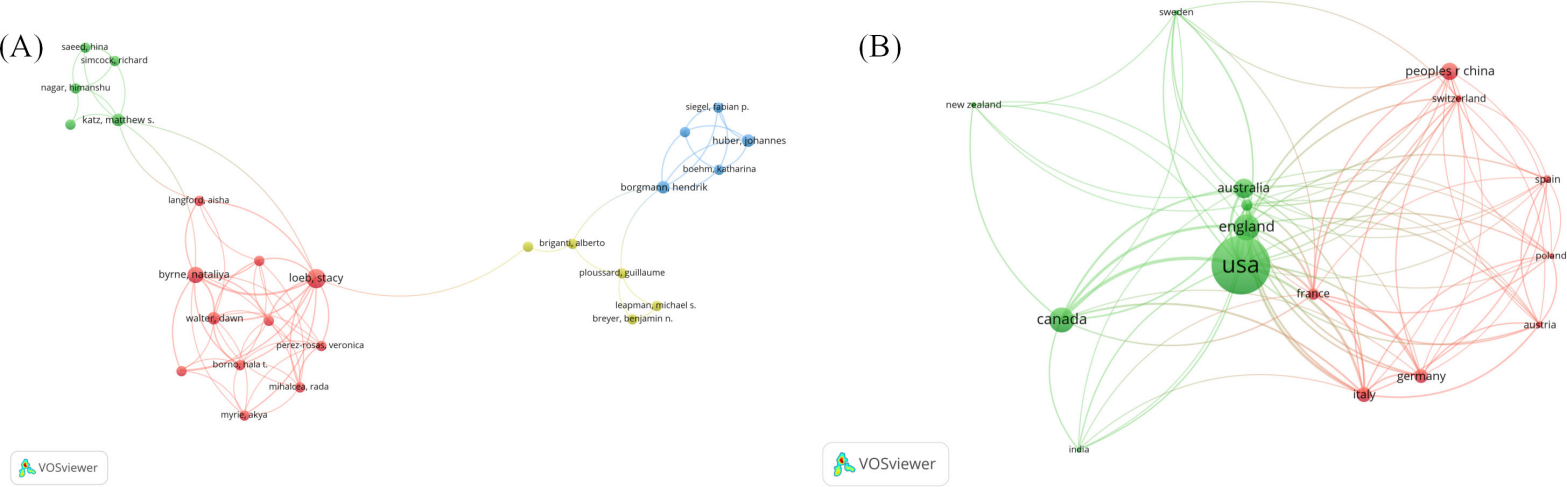
Collaborative network analysis of prostate cancer research on social media platforms (**(A)** Author level; **(B)** Country level).

The collaborative network map (**Figure 1**) was generated using VOSviewer. The node size represents the number of publications, assessed by the Total Link Strength (TLS) metric. In bibliometric analysis, TLS quantifies the strength of connections between items.

In the author collaborative network (**Figure 1A**), a map was generated from 26 sources, revealing four distinct clusters represented by different colours. Among the authors, Loeb S exhibited the highest TLS of 26, followed by Byrne N (22) and Walter D (16).

For the country-level collaborative network analysis (**Figure 1B**), the network comprised 19 nodes. The United Kingdom had the highest TLS of 66, followed by the United States (64) and Italy (43). The network formed two coloured clusters: one collaborative group consisted of the United States, the United Kingdom, Canada, and other countries, while another collaborative cluster included China, Switzerland, Italy, and others. Based on node size, the United States possessed the largest node.

### Citation analysis

**Supplementary Table 1** lists the top 10 most cited articles in the field of social media-based prostate cancer research. The study by Loeb S et al., titled ‘Dissemination of Misinformative and Biased Information about Prostate Cancer on YouTube’ and published in European Urology in 2019, ranked first in both total citations (238) and average citations per year (34.0). The scoping review by Plackett R et al., ‘Use of Social Media to Promote Cancer Screening and Early Diagnosis: Scoping Review’ published in the Journal of Medical Internet Research in 2020, demonstrated a high normalized citation impact (Normalized TC, 2.71).

The 2019 study by Loeb S et al. was the first large-scale, systematic evaluation of prostate cancer-related content on YouTube, utilizing standardized tools like DISCERN and PEMAT to ensure objective assessment. Its core finding revealed a paradox between information quality and dissemination: videos containing scientifically inaccurate or biased information (77%) garnered higher user engagement and broader reach, indicating that popular videos are not necessarily reliable sources of information (22).

The 2020 scoping review by Plackett et al. highlighted that a significant proportion of included studies focused on social media interventions for prostate cancer (7/23, 30%), often mentioned alongside testicular cancer. Interventions were primarily conducted on platforms like Twitter (57%), frequently as part of national cancer awareness months (70%). The language used in prostate cancer-related social media content was often gendered, employing terms like ‘manly’ to construct masculinity in campaigns such as Movember and utilizing war metaphors (eg, ‘army’) to enhance user engagement. Furthermore, despite the existence of a dedicated prostate cancer awareness month (November), mentions of breast cancer on social media during the same period (284, 015 mentions) significantly outweighed those of prostate cancer (65, 820 mentions). The study also noted that tweets related to prostate cancer were more often associated with fundraising than with disseminating health information, with only 2% of fundraising tweets explicitly mentioning prostate or testicular cancer (23).

Related research primarily focuses on three fields: the quality assessment of health information on social media platforms related to prostate cancer and the analysis of its dissemination characteristics—such as the prevalence of misinformation (24), content bias (22) and the relationship between user engagement and information quality (25); the application models and effectiveness of social media in prostate cancer patient support (26), professional education (27) and public health interventions—including comparisons of online support groups (23), the operation of professional journal clubs (28) and evaluations of screening promotion campaigns (29); and methodological innovations and clinical integration exploration of emerging technologies (such as machine learning and NLP) in the analysis of medical data from social media (30).

**Figure 2:**
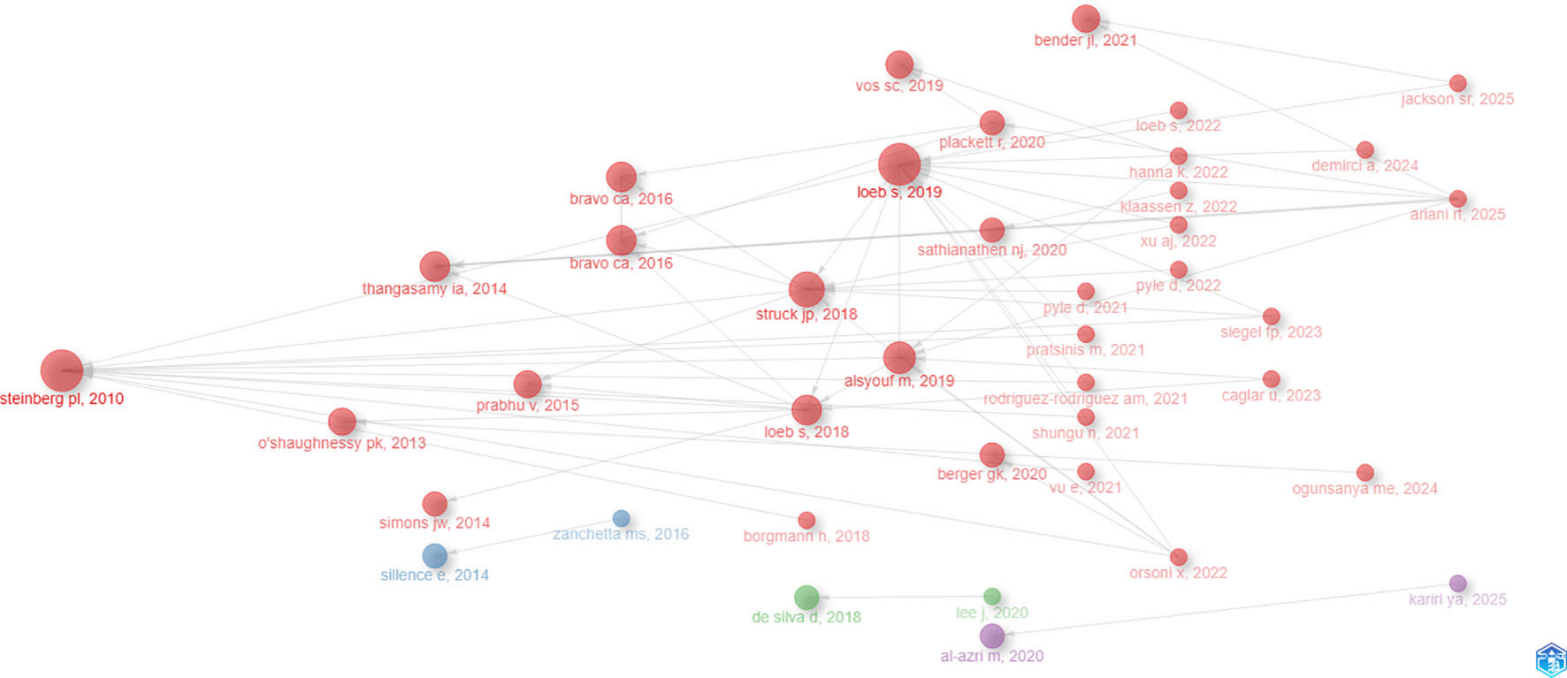
Citation pathways of publications related to prostate cancer research on social media platforms.

Further analysis of the citation pathways within the literature (**Figure 2**, generated by R package bibliometrix) reveals the existence of highly focused citation clusters, with the 2010 study by Steinberg et al. serving as a central node. This work has significantly shaped the evolution of research exploring social media in the context of prostate cancer.

The study by Steinberg PL et al. (2010) provided the first systematic evaluation of the quality and reliability of YouTube as a source of information on prostate cancer. By analysing 51 popular videos concerning PSA testing, radiotherapy, and surgery for prostate cancer, the study established a methodological framework for assessing information content and bias. A key finding was that the vast majority (73%) of the videos were of moderate or poor informational quality, and a significant proportion (69%) exhibited bias towards promoting specific tests or treatments, while largely failing to mention alternative management options such as active surveillance (25). The critical importance of this seminal study lies in its use of empirical data to highlight the potential risks of social media platforms as sources of health information, demonstrating that highly popular videos are not necessarily accurate or comprehensive. It set a methodological benchmark for subsequent research and established the core focus of this field on information quality and bias.

These citation pathways reflect a logical progression from initial research validating the quality of prostate cancer information towards more complex investigations into its behavioural impact, technological innovations, and clinical integration, thereby constructing a robust knowledge architecture for future studies.

### Analysis of research hotspots

Keywords represent a highly condensed summary of the core research content by authors. Therefore, analysing the interrelationships between keywords allows for the objective and efficient identification of widely recognized core topics, knowledge structures, and evolutionary trends within a specific research field.

**Figure 3:**
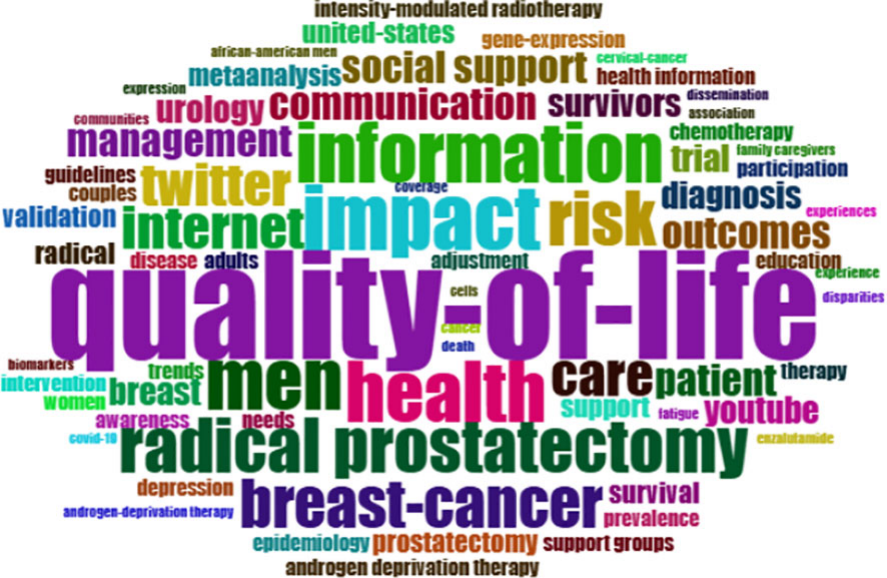
Word cloud of author keywords in prostate cancer research on social media platforms.

**Figure 3** presents a word cloud diagram generated from a R package bibliometrix analysis, displaying the 70 most frequent author keywords in the field of social media-based prostate cancer research. The high-frequency keywords include ‘impact’, ‘quality of life’, ‘health’, ‘information’, ‘men’, ‘radical prostatectomy’, ‘risk’, ‘care’, ‘internet’, ‘quality’, ‘Twitter’ and ‘communication’, among others.

**Figure 4:**
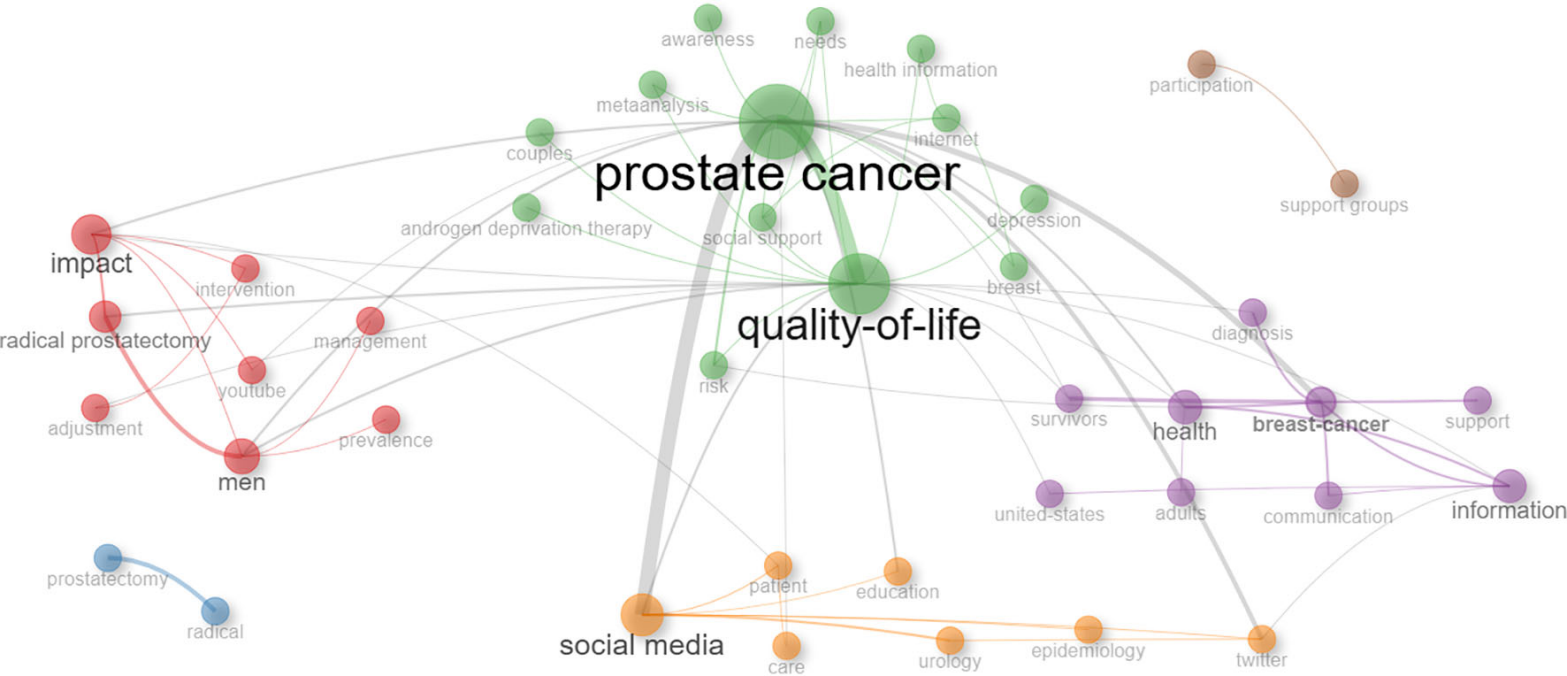
Co-occurrence clustering network of keywords in prostate cancer research on social media platforms.

Based on the identification of high-frequency keywords, a keyword co-occurrence cluster analysis was subsequently performed to explore the thematic structure and relationships between these terms. This analysis, visualized in **Figure 4**, revealed four distinct major clusters, each represented by nodes and connecting lines of a specific colour, indicating different primary research themes within the field.

The green cluster focuses on the comprehensive needs and support systems for patients with prostate cancer. Representative keywords include ‘prostate cancer’, ‘awareness’, ‘needs’, ‘health information’, ‘meta-analysis’, ‘couples’, ‘androgen deprivation therapy’, ‘social support’, ‘internet’ and ‘depression’.

The red cluster centres on the impact of radical prostatectomy on male patients and associated post-operative management interventions. Key terms in this cluster are ‘impact’, ‘radical prostatectomy’, ‘men’, ‘adjustment’, ‘prevalence’, ‘management’, ‘intervention’ and ‘YouTube’.

The yellow cluster revolves around patient education for prostate cancer through social media. This theme is characterized by keywords such as ‘social media’, ‘patient’, ‘education’, ‘care’, ‘urology’, ‘epidemiology’ and ‘Twitter’.

The purple cluster highlights comparative or benchmark studies, focusing on the governance of health information and patient education for breast cancer on social media platforms, with implications for prostate cancer research. Keywords forming this cluster include ‘breast cancer’, ‘health’, ‘information’, ‘survivors’, ‘support’, ‘adults’, ‘communication’ and ‘United States’.

**Figure 5:**
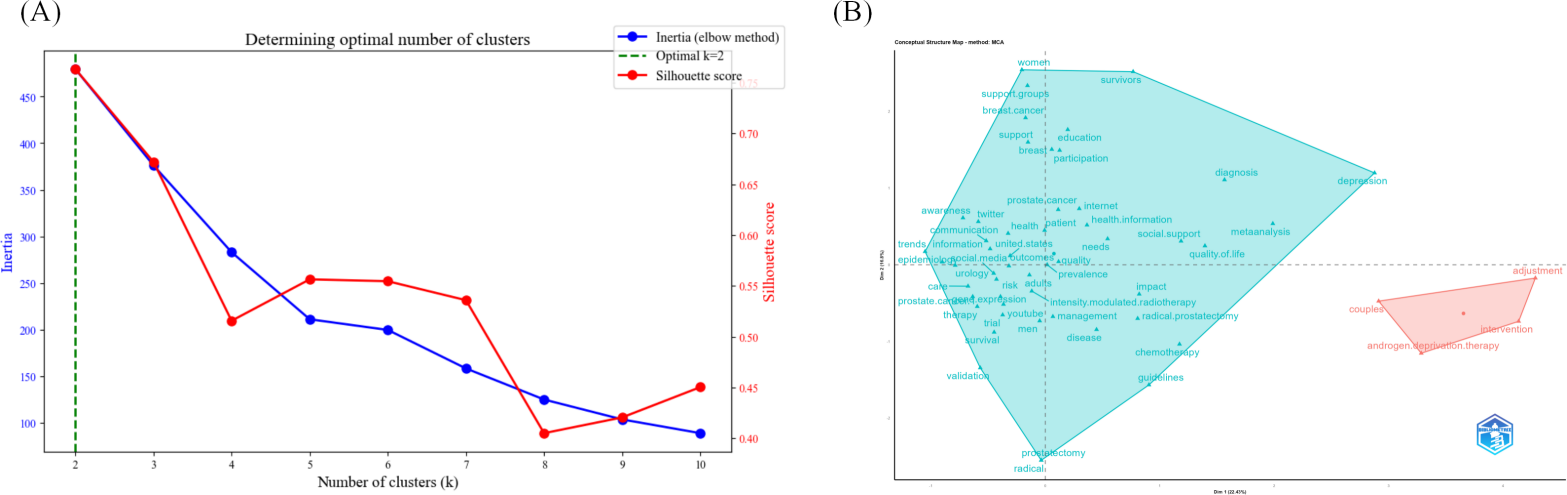
Factor analysis of keywords in prostate cancer research on social media platforms. **(A)** Determining optimal number of clusters; **(B)** Conceptual structure map.

**Figure 6:**
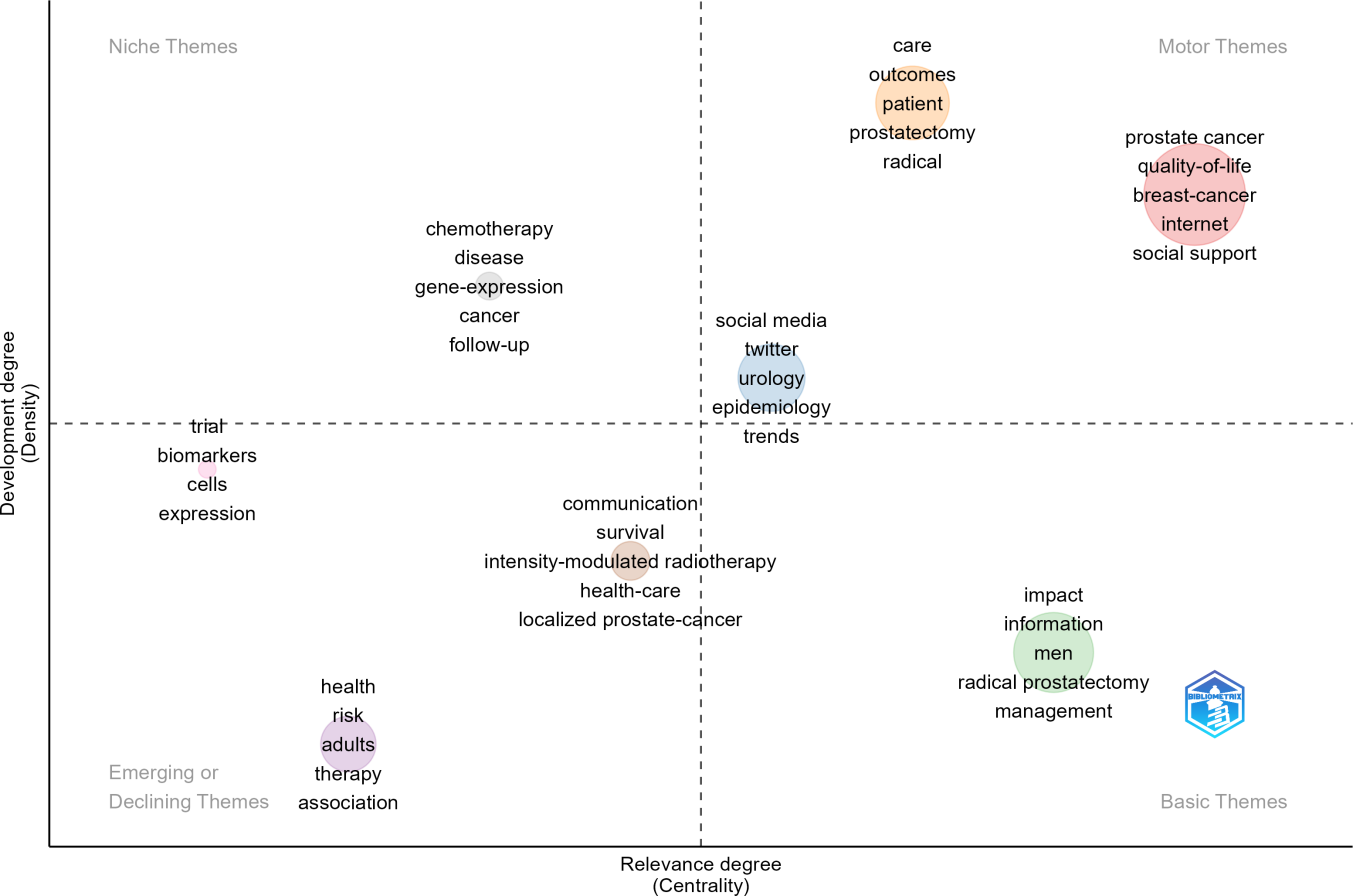
Thematic map based on density and centrality in prostate cancer research on social media platforms.

Building upon the keyword clusters identified in the current research on prostate cancer via social media platforms, a factor analysis (**Figure 5**) was conducted to extract underlying factors, aiming to reveal the reasons driving the divergence in research perspectives. As shown in **Figure 6**, factor 1 (represented by the red cluster) is highly loaded with keywords such as ‘adjustment’, ‘couples’ and ‘intervention’. This indicates a research perspective that closely focuses on the psychosocial impact of clinical treatments (eg, prostatectomy) on individuals and their intimate relationships, and explores corresponding adaptive intervention strategies via social media platforms. Factor 2 includes a broader set of keywords such as ‘prostate cancer’, ‘social media’, ‘breast cancer’, ‘survivors’, ‘quality of life’, ‘Twitter’ and ‘information’. This highlights how the technological attributes of social media shape information dissemination patterns for prostate cancer, positioning these platforms as a comprehensive ecosystem for health information dissemination and patient support.

In addition to keyword co-occurrence and cluster analysis, a thematic map (**Figure 6**) was generated using R package bibliometrix to visualize the structural landscape and developmental potential of research themes within the field of social media and prostate cancer. In this map, the horizontal axis (centrality) indicates a theme’s relevance and connection to the broader research network, while the vertical axis (density) represents the internal development and cohesion of the theme. The four quadrants categorize themes into distinct developmental directions.

‘Motor Themes’ located in the upper-right quadrant (eg, ‘care’, ‘outcomes’, ‘quality of life’, ‘social media’) indicate that a comprehensive research system focusing on patient outcomes and internet-based support has been established in this field. ‘Niche Themes’ in the upper-left quadrant (eg, ‘chemotherapy’, ‘gene expression’), while having lower centrality, exhibit mature internal development, representing highly specialized research directions. ‘Basic Themes’ in the lower-right quadrant (eg, ‘information’, ‘management’) are highlighted by their high centrality, underscoring key horizontal concepts that permeate the entire field. Finally, ‘Emerging or Declining Themes’ in the lower-left quadrant (eg, ‘biomarkers’, ‘intensity-modulated radiotherapy (IMRT)’) reflect current frontier explorations that, while still peripheral, hold potential for future development.

**Figure 7:**
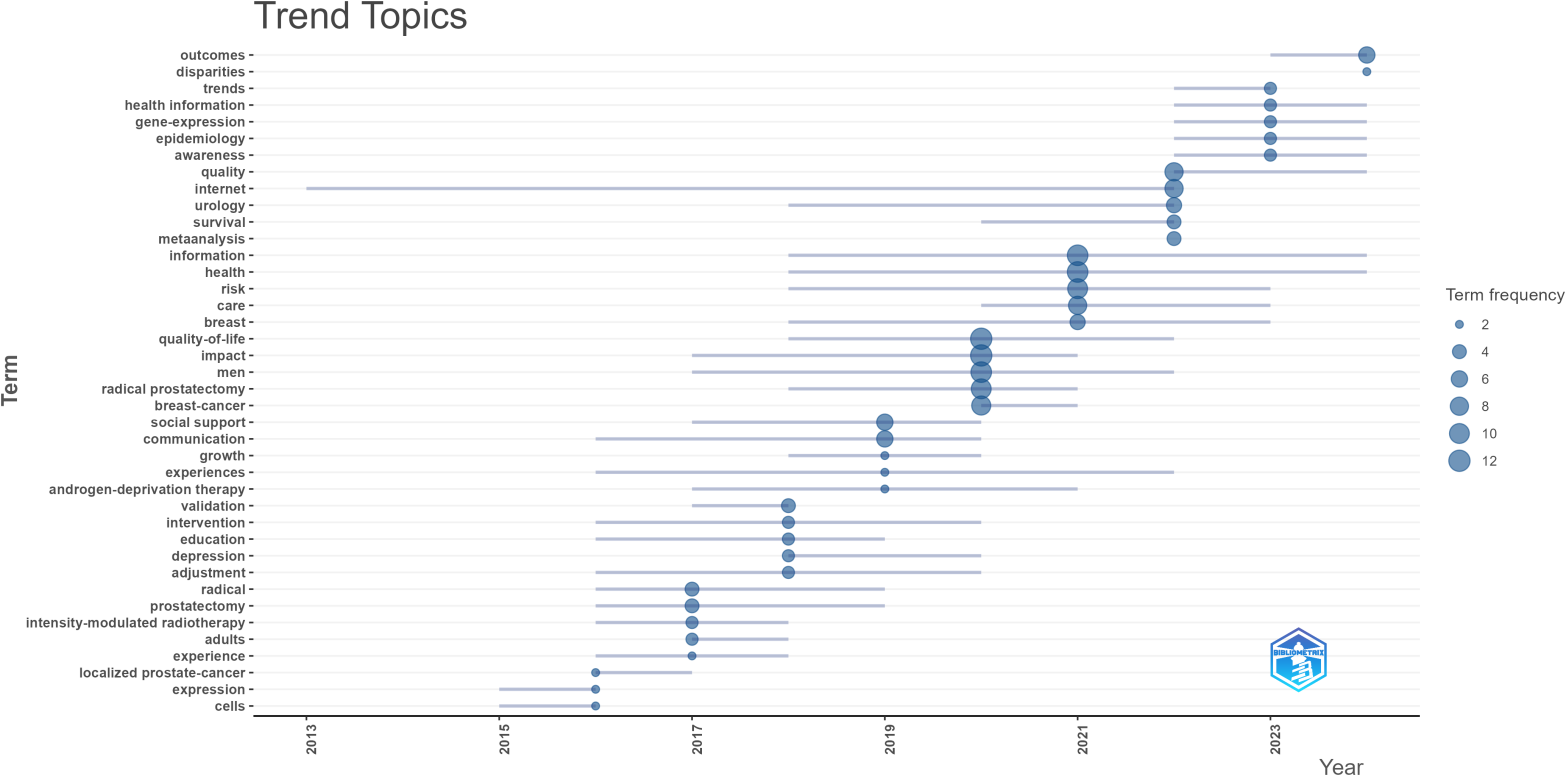
Evolution of trending topics based on keywords in prostate cancer research on social media platforms.

The Trend Topic Map (**Figure 7**) illustrates the distribution of keywords identified based on burst strength across different time periods, revealing three distinct stages in the evolution of research foci within social media-based prostate cancer studies.

In the period prior to 2016, prominent keywords such as ‘localized prostate-cancer’, ‘expression’ and ‘cells’ primarily reflected a research focus on fundamental disease biology and underlying mechanisms.

A significant expansion of research scope occurred between 2017 and 2019. During this phase, a multitude of keywords emerged, including ‘social support’, ‘communication’, ‘experiences’, ‘radical prostatectomy’ and ‘intensity-modulated radiotherapy’. This shift demonstrated a marked diversification of interest towards psychosocial aspects of patient care, social support systems, and specific treatment modalities.

The period from 2020 to 2022 saw the rise of keywords like ‘quality-of-life’, ‘internet’, ‘care’, ‘information’, ‘survival’ and ‘meta-analysis’. This evolution signified a transition from a primary focus on psychological intervention towards a broader emphasis on overall survival quality. Furthermore, the perspective expanded from the individual level to encompass macro-level public health considerations, integrating evidence-based medicine, risk factor analysis, and health information dissemination.

From 2023 onwards, burst keywords such as ‘trends’, ‘health information’ and ‘gene-expression’ have appeared, with the most recent data from 2025 highlighting ‘outcomes’ and ‘disparities’. This suggests the research frontier is now advancing towards health informatics, epidemiological trends and studies addressing health equity.

Overall, the field demonstrates a clear trajectory of evolution, progressing from basic biological inquiry to patient-centred and psychosocial support research, and further towards addressing broader public health issues.

## Discussion

### General characteristics

This study presents the first bibliometric analysis of the research landscape concerning prostate cancer on social media platforms, systematically delineating its evolution, distribution, and collaborative networks. The annual publication output demonstrates a general upward trend, with seminal studies in specific years exerting a dominant influence on the overall research trajectory. High-yield academic publications are predominantly concentrated in authoritative journals within urology and men’s health. Regarding author contributions, Loeb S emerges as the most influential scholar in this domain. Regarding national contributions, the United States leads in productivity, while the United Kingdom occupies a central role in international collaborations, a position likely associated with its sustained research investment and early strategic focus (31). Countries such as Australia and Canada also demonstrate considerable research strength and international collaborative capacity. However, the research landscape shows limited representation from developing countries, indicating a future need for enhanced global resource integration and research cooperation.

### Research priorities and implications

Keyword analysis indicates that current research hotspots focus on leveraging social media for health information quality assessment, patient education, and delivering personalised patient support and quality-of-life interventions.

Health information quality assessment primarily concerns the accuracy, reliability, and actionability of the vast amount of health content on social media. This issue is particularly pronounced in the context of prostate cancer research. For instance, Loeb et al.’s 2019 study found that approximately 77% of misleading prostate cancer information videos on YouTube paradoxically received higher user engagement (22). Another study focusing on Instagram revealed that up to 90% of content related to prostate cancer on the platform was of low to medium quality, with only a very small proportion (9%) created by physicians (32). The proliferation of misinformation can lead to inappropriate treatment choices, thereby affecting patient outcomes (33). Furthermore, misinformation can negatively impact patients’ psychological well-being. Exaggerated descriptions of treatment side-effects may cause excessive anxiety during decision-making, affecting patients’ confidence in their choices (34). Consequently, there is a need for government departments to strengthen oversight of social media platforms, rigorously verify the professional credentials of content creators, and for platforms to assume greater responsibility in vetting information. Additionally, developing intelligent content aggregation systems and standardised information frameworks is crucial to ensure the accuracy and systematic nature of health content, thereby effectively countering misinformation.

Conducting patient education is another key focus of prostate cancer research on social media platforms. Compared to traditional educational models, social media, with its immediacy, broad accessibility and cross-platform nature, can effectively overcome geographical barriers to provide round-the-clock information support, demonstrating significant advantages. Current research has evolved from simple information dissemination towards exploration of more targeted, personalised education. For example, Alsyouf et al. (2019) effectively increased public awareness of prostate cancer genetic risk and genetic testing by creating and testing specific social media messages (24). The potential of this approach lies in its ability to tailor content according to the patient’s disease stage, cultural background and information preferences, thereby significantly enhancing engagement and effectiveness of education (35). Furthermore, artificial intelligence (AI) technologies, particularly large language models (LLMs), create opportunities for deepening personalised education (36). Research suggests that such tools can extract key information from massive amounts of unstructured online dialogue, accurately identifying patients’ knowledge gaps and concerns, thus providing data support for developing highly personalised educational content strategies (37). However, it is important to note that prostate cancer patient education on social media platforms still faces challenges such as fragmented educational content and uneven quality, which may compromise the ultimate effectiveness of education (38). For instance, an analysis of prostate cancer screening videos on YouTube showed that although most videos (71.1%) mentioned potential harms of screening, the overall information quality still needed improvement (39). Additionally, on Instagram, most prostate cancer-related content was of low to medium quality and had poor actionability (32). This highlights the need to encourage greater participation by healthcare professionals in disseminating high-quality information on social media to address potential misinformation issues.

Notably, keyword analysis reveals a significant volume of ‘breast cancer’-related research within the social media-focused prostate cancer literature, highlighting several important characteristics and the background of this research field. As a highly prevalent cancer in men (prostate cancer) and a highly prevalent cancer in women (breast cancer), these two diseases are often studied comparatively to explore the similarities and differences in online community activity, support models, and awareness campaign effectiveness (40). However, compared to the successful experience of breast cancer in utilizing social media for public education, fundraising and patient support (eg, the ‘Pink Ribbon’ campaign (41)), the use of social media for patient education in prostate cancer appears relatively less optimistic. A population-based survey in the UAE showed that breast cancer was the most frequently mentioned cancer in the media (72.7%), while the mention rate of prostate cancer was only 2.1%, reflecting a structural imbalance in gendered health narratives within health discourse (42). This phenomenon may stem from multiple factors: traditional societal expectations of masculinity often inhibit men’s willingness to openly discuss health issues (43); the mature organizational models and visual symbols (eg, the pink ribbon) formed by breast cancer advocacy movements have garnered higher public visibility (41); concurrently, the unequal distribution of research resources and public attention among different diseases is also an important reason (44). This suggests that future research should delve into how to strategically adapt the successful experiences of the breast cancer community for the prostate cancer patient population. Secondly, developing health communication strategies with greater gender sensitivity is needed to overcome socio-cultural barriers surrounding men’s health discussions. Finally, increased policy-level attention and funding support are essential to promote a more balanced public awareness system for cancer prevention and control, thereby addressing the current health information inequality.

Utilizing social media platforms for patient support and quality of life interventions for prostate cancer patients primarily focuses on user needs identification, intervention efficacy evaluation, and social interaction/psychological support. For instance, Thompson et al. conducted a meta-analysis of the cross-diagnostic Internet Delivery Acceptance Commitment Therapy (iACT) and found that it significantly improved anxiety, depression and quality of life in various clinical populations (45). Additionally, interventions employing Acceptance and Commitment Therapy (ACT), utilizing online diaries and feedback, help patients accept disease uncertainty and reduce avoidant coping (46). Online support groups and peer interaction also serve as a valuable method. One study found that such interventions, aimed at managing emotions like anger and depression arising from the disease and its treatment through organized discussions, can act as a bridge connecting inpatient care with daily life (47). Future research priorities should concentrate on optimizing intervention strategies to ensure their effectiveness and exploring how to more widely integrate successful support models into routine care.

### Research frontiers and future directions

Analysis of thematic and trend maps indicates that social media-based prostate cancer research is undergoing a paradigm shift, moving from micro-level individual care towards macro-level precision monitoring. Utilising social media platforms for disease prediction represents an emerging frontier. Future development will deeply integrate AI technologies with the principles of precision medicine, while simultaneously striving to address existing challenges related to equity and data quality.

AI, particularly NLP and LLMs, is reshaping the paradigm of disease trend monitoring. These technologies can integrate multi-source data, extracting valuable insights from unstructured social media content, patient forum discussions, and even clinical radiology reports. For instance, machine learning models analysing patient-generated online discussions can identify unmet clinical needs (48), regional variations in diagnosis and treatment (49), and even assess public awareness of specific knowledge, such as BRCA genetic testing (50). This enables dynamic and precise monitoring of disease trends, diagnostic and treatment patterns, and the impact of public health events on prostate cancer care pathways. Furthermore, the research community is placing increasing emphasis on health equity. Using AI to analyse discussion content and information accessibility across different racial, geographic, and socioeconomic groups on social media helps reveal disparities in the prostate cancer burden, informing the development of more targeted public health interventions (50). Additionally, researchers are beginning to explore using social media for genetic testing literacy and precision risk communication, and to evaluate the impact of advanced treatment technologies on patient quality of life and survival outcomes (50, 51).

AI digital humans and chatbots also hold significant potential as virtual health assistants, providing patients and their families with accurate and easy-to-understand information, providing patients and their families with accurate, easy-to-understand information 24/7 (51), such as explaining complex treatment options (52), managing side effects (53), or follow-up care instructions (54). They can offer personalised information based on a patient’s diagnostic stage, treatment plan, and knowledge level. Moreover, through sustained, human-like interactions, reminders, and emotional support, AI digital humans can potentially support better treatment adherence compared to traditional information dissemination methods (55). For example, an educational intervention using an AI chatbot named EpiloBot for epilepsy patients showed statistically significant improvements in patients’ knowledge, attitudes, and reduced stigma scores (56). On the other hand, the interactive nature of chatbots makes them effective tools for dynamic data collection. While ensuring privacy protection, they can anonymously record frequently asked questions and knowledge gaps, providing real-time interaction data that can feed back into macro-level public health strategies, enabling precise needs assessment and intervention (57).

Despite the considerable potential, significant challenges remain in social media-based prostate cancer research. A prominent issue is that, compared to cancers like breast cancer, prostate cancer generally receives lower overall attention and media mention on social media (58). This attention gap may introduce data bias, affecting the comprehensiveness and representativeness of trend monitoring (59). Furthermore, AI models can be susceptible to data inaccuracies, and technical challenges in multi-modal data alignment pose additional demands on the accuracy of monitoring results (60). Future research needs to focus on developing more robust algorithms and actively promoting interdisciplinary collaboration between clinicians and data scientists to build fairer, more reliable, and efficient intelligent trend monitoring systems for prostate cancer.

### Limitations

This study has several limitations. First, the inclusion of only English-language publications may have led to the omission of valuable research published in other languages, thereby affecting the comprehensiveness and representativeness of the findings. Second, the selection of node parameters (eg, thresholds for clustering algorithms, length of time slices) involved a degree of subjectivity. Different researchers, depending on their specific research objectives or interpretative perspectives, might choose divergent parameters, which could introduce analytical bias. In addition, the choice of platforms used in this study was constrained by our institutional resources and database access conditions. Future studies could enhance the breadth, depth, and accuracy of findings by incorporating non-English literature, employing mixed-methods approaches, and additional databases (e.g., Scopus). Meanwhile, the application of multi-parameter settings and cross-validation techniques can help improve the reliability and comparability of the results.

## Conclusions

This study, leveraging data from the WoS, PubMed, and IEEE Xplore databases, employed bibliometric and thematic analysis to identify prolific authors, countries, and journals within the field of social media-based prostate cancer research. It has systematically delineated the current state of knowledge, intellectual structure, and evolutionary trends in this domain. The aim is to provide researchers, clinicians, and policy-makers with an evidence-based overview of the field. The findings offer an evidence-informed basis for understanding the current landscape and future potential of social media in prostate cancer research, which can inform strategic planning, foster interdisciplinary collaboration, and guide the prioritisation of resources.

## Data Availability

The original contributions presented in the study are included in the article/**Supplementary Material**. Further inquiries can be directed to the corresponding author.
