# Supplementary material for: Prostate cancer research on social media platforms: a bibliometric and thematic analysis
Source: Front Oncol. 2026 Jun 2;16:1799912. doi: 10.3389/fonc.2026.1799912 (PMC13268919; doi:10.3389/fonc.2026.1799912)
Supplement: Supplementary file 1 [file Table1.docx]

**Supplementary** **Table 1.** Top-ranked articles by citation count in social media-based prostate cancer research

| **Rank** | **Paper** | **DOI** | **Total Citations** | **TC per Year** | **Normalized TC** |
| --- | --- | --- | --- | --- | --- |
| 1 | LOEB S, 2019, EUR UROL | 10.1016/j.eururo.2018.10.056 | 238 | 34.00 | 1.49 |
| 2 | STEINBERG PL, 2010, UROLOGY | 10.1016/j.urology.2008.07.059 | 203 | 12.69 | 1.00 |
| 3 | THANGASAMY IA, 2014, EUR UROL | 10.1016/j.eururo.2014.01.034 | 120 | 10.00 | 2.21 |
| 4 | HUBER J, 2018, J CANCER SURVIV-RES PRACT | 10.1007/s11764-017-0633-0 | 91 | 11.38 | 2.60 |
| 5 | ALSYOUF M, 2019, BJU INT | 10.1111/bju.14787 | 90 | 12.86 | 0.56 |
| 6 | PLACKETT R, 2020, J MED INTERNET RES | 10.2196/21582 | 74 | 12.33 | 2.71 |
| 7 | DE SILVA D, 2018, PLOS ONE | 10.1371/journal.pone.0205855 | 51 | 6.38 | 1.46 |
| 8 | STRUCK JP, 2018, WORLD J UROL | 10.1007/s00345-018-2254-2 | 44 | 5.50 | 1.26 |
| 9 | ZHANG L, 2018, INT J MED INFORM | 10.1016/j.ijmedinf.2018.10.002 | 44 | 5.50 | 1.26 |
| 10 | BRAVO CA, 2016, J CANCER EDUC | [10.1007/s13187-015-0796-1](https://doi.org/10.1007/s13187-015-0796-1) | 36 | 3.27 | 1.93 |
