# Supplementary figures and images for: Prostate cancer research on social media platforms: a bibliometric and thematic analysis
Source: Front Oncol. 2026 Jun 2;16:1799912. doi: 10.3389/fonc.2026.1799912 (PMC13268919; doi:10.3389/fonc.2026.1799912)

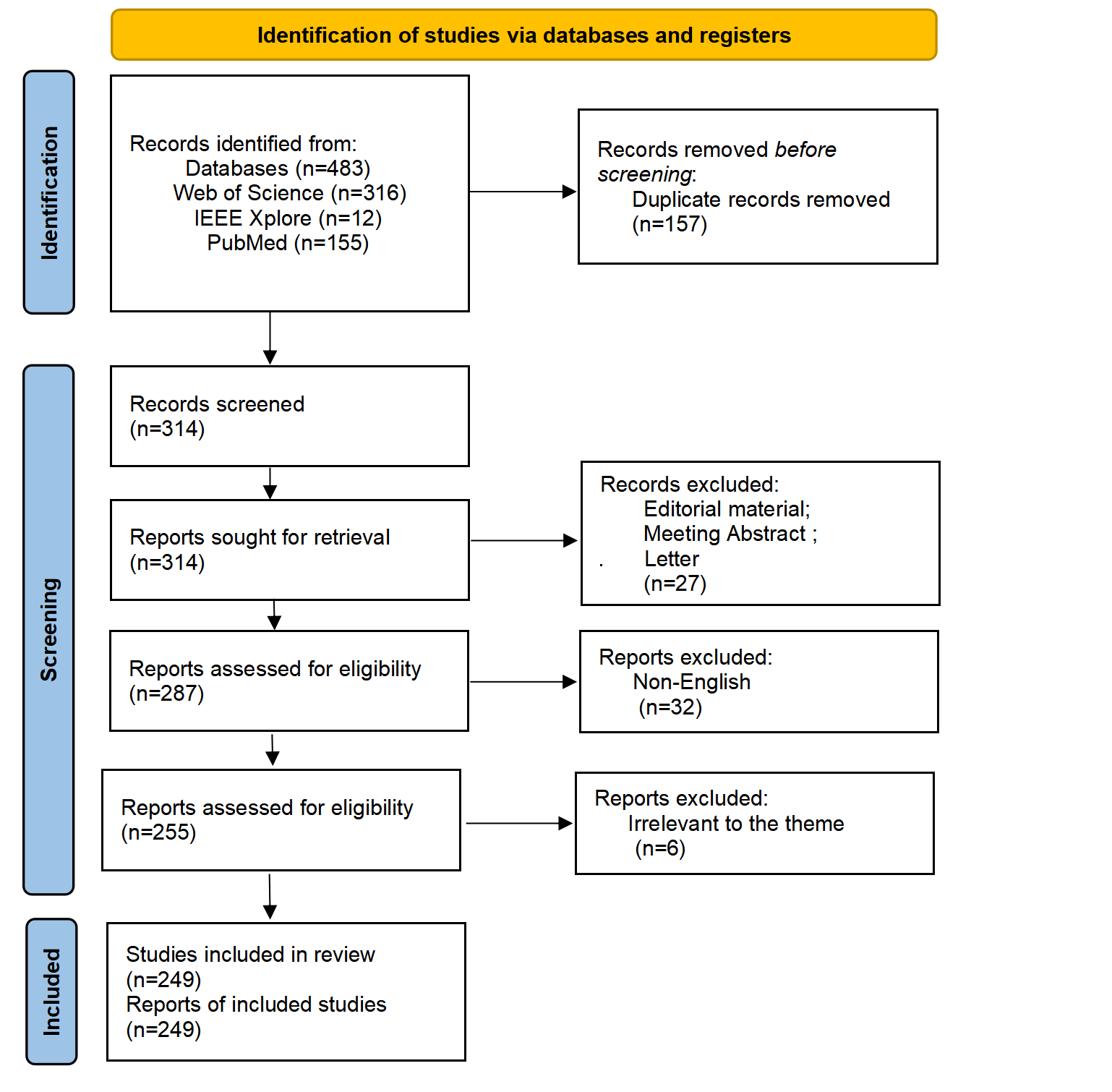

Supplement: Supplementary file 2 [file Image1.tif]

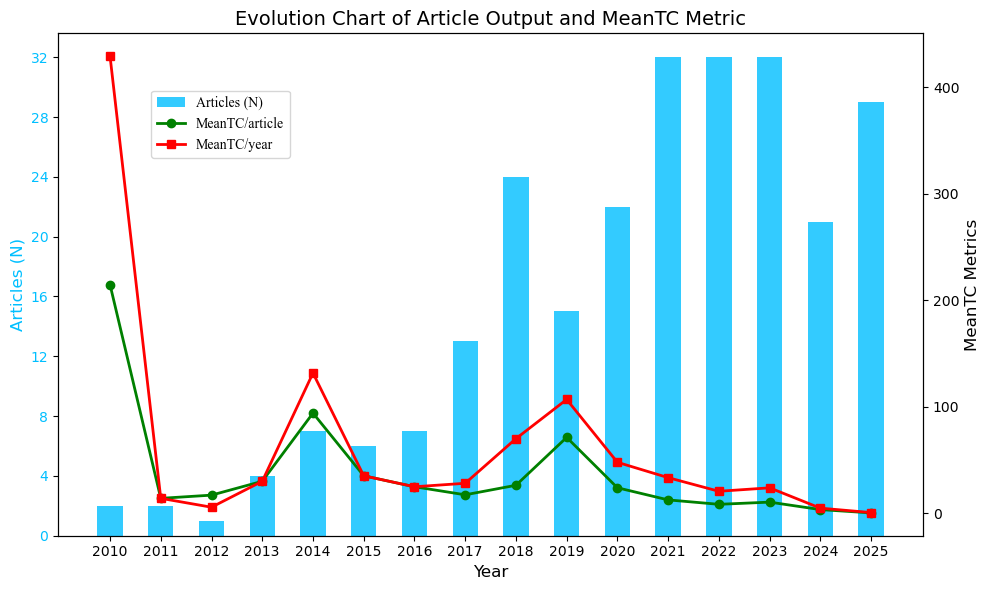

Supplement: Supplementary file 3 [file Image2.png]
